# The antipredator benefits of postural camouflage in peppered moth caterpillars

**DOI:** 10.1038/s41598-020-78686-4

**Published:** 2020-12-10

**Authors:** Hannah M. Rowland, Robert P. Burriss, John Skelhorn

**Affiliations:** 1 Max Planck Research Group Predators and Toxic Prey, Max Planck Institute for Chemical Ecology, Hans Knӧll Straβe 8, 07745 Jena, Germany; 2 Department of Zoology, University of Cambridge, Downing Street, Cambridge, CB2 3EJ UK; 3 Department of Psychology, The University of Basel, Missionsstrasse 62, 4055 Basel, Switzerland; 4 Biosciences Institute, Faculty of Medical Sciences, Newcastle University, Henry Wellcome Building, Framlington Place, Newcastle upon Tyne, NE2 4HH UK

**Keywords:** Evolution, Behavioural ecology, Ecology, Entomology, Animal behaviour

## Abstract

Camouflage is the most common form of antipredator defense, and is a textbook example of natural selection. How animals’ appearances prevent detection or recognition is well studied, but the role of prey behavior has received much less attention. Here we report a series of experiments with twig-mimicking larvae of the American peppered moth *Biston betularia* that test the long-held view that prey have evolved postures that enhance their camouflage, and establish how food availability and ambient temperature affect these postures. We found that predators took longer to attack larvae that were resting in a twig-like posture than larvae resting flat against a branch. Larvae that were chilled or food restricted (manipulations intended to energetically stress larvae) adopted a less twig-like posture than larvae that were fed ad libitum. Our findings provide clear evidence that animals gain antipredator benefits from postural camouflage, and suggest that benefits may come at an energetic cost that animals are unwilling or unable to pay under some conditions.

## Introduction

Much is known about how natural selection has shaped the appearance of camouflaged animals^1–3^. In contrast, we know considerably less about *behavioral camouflage*—the role that the behavior of camouflaged animals plays in determining how difficult they are to locate and identify^4^. There are many ways in which an animal’s behavior could influence the extent of its camouflage. Cryptic animals often choose to rest against backgrounds that best match their own color/pattern^4–7^, and can orient themselves in a manner that improves this match and further reduces detectability^8^. Animals that masquerade as inedible objects (e.g. twigs, stones and leaves) are known to select microhabitats in which examples of their inedible models are both common^9^ and good matches for their own appearance^10,11^. Both cryptic and masquerading animals can modify their own appearance^12,13^ or that of the background^14,15^ in a manner that appears to make them more difficult to detect or recognize.

It has also been suggested that many animals could benefit from *postural camouflage*^16,17^: holding their bodies or body parts in postures that make them more difficult to detect or correctly identify (note that this is distinct from how they position themselves against the background e.g. Ref.^4^). For example, several species of cryptic lizard rest flattened against a substrate with their limbs drawn in along the body axis^18,19^; many insects protract their legs against—and parallel to—the long axis of the body, concealing their head and antennae and creating a more linear appearance^20^. In addition to this, the postures adopted by masqueraders often appear to increase their resemblance to inedible objects^16,21^. Potoos rest with their beaks raised and their eyes closed, making them appear more similar to a branch stump^22^; the sea slug Aplysia *punctata* (family Aplysiidae) rest with their bodies extended and their tentacles and pleuropodia arranged in a manner that seems to simulate stunted branches of weed^17^; and cephalopods alter both their texture and the position of their arms such that their resemblance to items as diverse as rocks, plants, and branching corals seems increased^23–25^.

**Figure 1:**
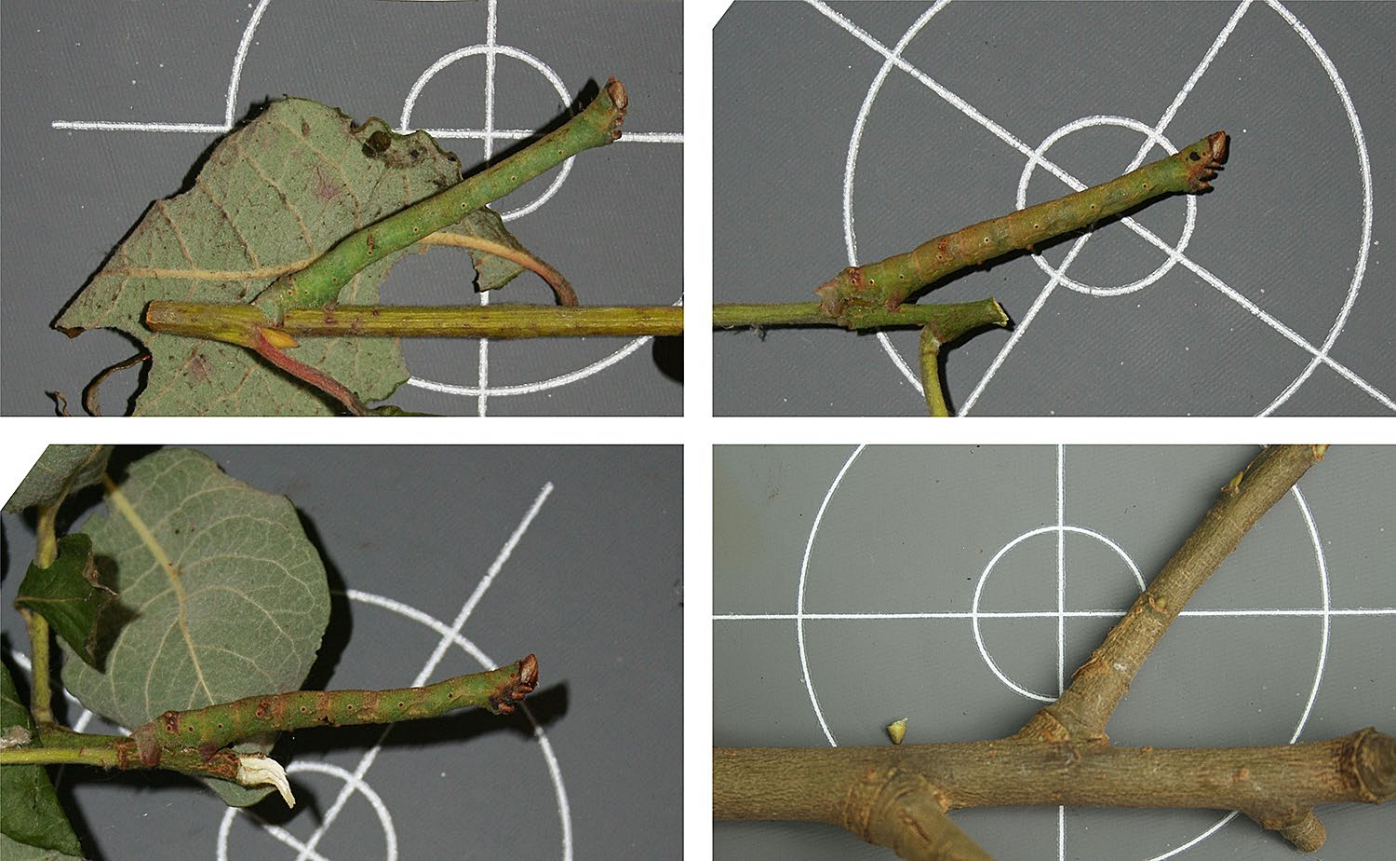
American peppered moth larvae resting in twig-like postures. The images are of the larvae with the median resting angle in each of the experimental conditions in Experiment 2: Ad lib (top left), chilled (bottom left), and food restricted (top right). The remaining image (bottom right) is for comparison, and represents the angle between branches and twigs from Experiment S1.

Despite references to postures that function to enhance camouflage dating back over 100 years^16^, there is very little empirical evidence that these examples of putative postural camouflage have an antipredator benefit^4,20,26^. There is tentative support for the idea that masquerading species adopt postures that make them appear more similar to their models^27^. Twig-mimicking larvae of the American peppered moth rest in a twig-like posture (see Fig. 1) at an angle that is similar to, although not quite as high as, the angle between twigs and the branches to which they are attached (Ref.^27^ and see SI Experiment 1). There is also some evidence that larvae that masquerade as bird droppings tend to adopt bent postures that appear to enhance this resemblance, and that pastry models of these caterpillars are less prone to predation when presented in a bent compared to a straight posture^26^. While this may be because the posture enhances caterpillars’ resemblance to bird droppings^26^, other interpretations of these data have been proposed^4^, and it remains unclear to what degree the model larvae accurately reflected either the appearance or the posture of real caterpillars.

While there is only limited empirical evidence that postural camouflage has an antipredator benefit, intraspecific variation in this behavior has received even less attention. If postural camouflage is associated with energetic costs (which is not necessarily the case^28^, but seems to be true of other forms of defensive posture^29^), then we might expect posture to be influenced by environmental conditions that impact an individual’s state. Here we exploit natural variation in the tendency of American peppered moth larvae to utilize twig-like postures (see SI experiment 2) to test whether naïve avian predators take longer to find and attack larvae when they are in these postures than when they are not (i.e., whether they benefit from postural camouflage). We then establish whether decreased temperature and food availability, two factors that typically reduce growth in caterpillars^30^, affect the resting postures of these twig mimicking caterpillars.

## Results

### Experiment 1: Does postural camouflage serve an antipredator function?

Thirty-two domestic chicks were trained to forage in an experimental arena before being randomly-assigned to one of two equally-sized experimental groups. Each chick was then given three consecutive trials in which it encountered a single larva presented on a 20 cm long willow branch with 8 twigs. The posture of the larvae differed between our experimental groups: one group encountered larvae resting in a twig-like posture, and the other encountered larvae resting flat against the branch. In each trial, chicks were allowed unlimited time to make their first attack on either a branch or a larva, and were then observed for a further 3 min to determine whether they ate the larva. We recorded whether chicks attacked the branch before they attacked the larva (i.e. whether they were error prone), the latency to find and attack the larva, and whether the larva was eaten.

**Figure 2:**
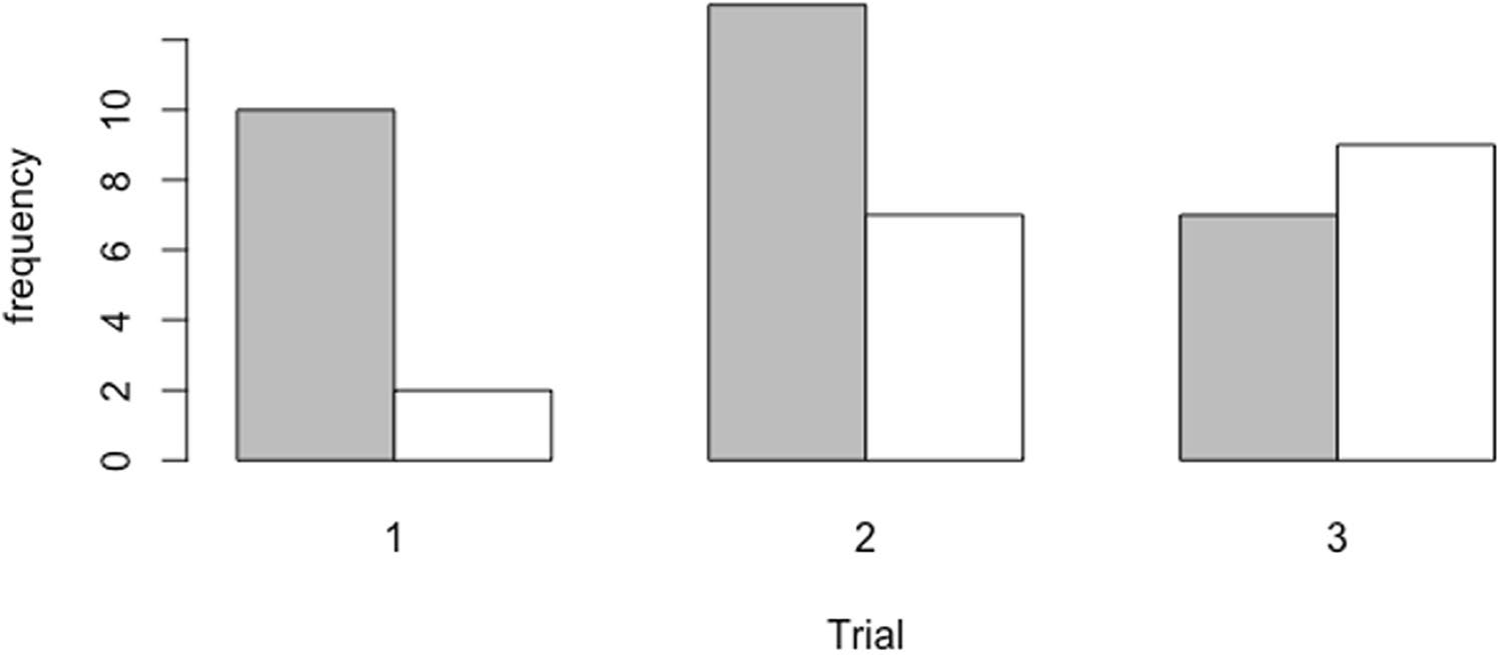
The frequency of larvae attacked first when presented flat (gray) or at a posture (white) across trials.

Chicks attacked twigs before they attacked larvae more often when larvae rested in a twig-like posture compared to when they rested flat (estimate = − 5.2875 ± 1.7327, z = − 3.052, *p* = 0.00228), and chicks presented with larvae in a twig-like posture were initially more error prone than those presented with larvae resting flat (treatment*trial interaction: estimate = 1.9061 ± 0.7144, z = 1.134, *p* = 0.00763; Fig. 2).

**Figure 3:**
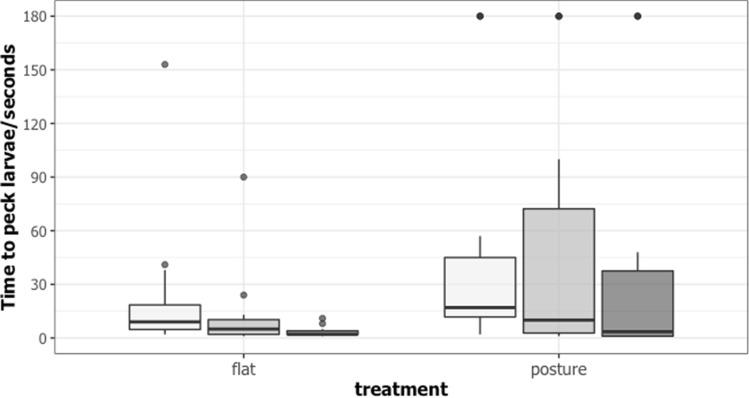
Latency to attack larvae in seconds for larvae presented flat or in a posture from trial 1–3 (light to dark gray).

Chicks presented with larvae resting at an angle also took significantly longer to find and attack larvae than those presented with larvae resting flat against the branch (estimate = 1.1274 ± 0.4408; z = 2.558, *p* = 0.0105; see Fig. 3) and the latency to attack the larvae decreased across trials (trial 1 vs. trial 2: estimate = − 0.5452 ± 0.2418; z = − 2.254, *p* = 0.24547; trial 1 vs. trial 3: estimate = − 1.1917 ± 0.2537; z = − 4.697, *p* = 2.64e−06). We found no evidence that the number of larvae eaten differed significantly between our experimental groups (estimate = 3.5059 ± 2.5038, z = 1.400, *p* = 0.161; in the posture group 56%, 44%, and 44% were eaten in trials 1–3, respectively, and in the flat group 69%, 69%, and 81%). Resting in a twig-like posture clearly makes larvae more difficult for predators to detect or recognize. However, predators’ ability to find and attack larvae improves with experience irrespective of larval posture.

### Experiment 2: Does food restriction and ambient temperature affect caterpillars’ posture?

Ninety-four final (5th) instar American peppered moth larvae were used to establish how decreased temperature and food availability affected caterpillars’ resting postures. These manipulations were intended to energetically stress caterpillars (see “Materials and methods” for justification). We reasoned that, if resting at an angle is energetically expensive, larvae that are hungry or cold will be less likely to rest at an angle than those that are not. Moreover, we predicted that if larger angles are more energetically expensive to hold, then cold and hungry larvae that rested at an angle should rest with their heads closer to the branch. We allocated size-matched larvae to three experimental groups: ad lib larvae (n = 28) were maintained at room temperature with a constant food supply, food restricted larvae (n = 31) were maintained at room temperature with no access to food for 48 h, and chilled larvae (n = 35) were refrigerated ($\approx$ 1–4 °C) but had access to a constant food supply for 48 h. After 48 h, larvae were transferred to a leafless branch and allowed to settle for 1 h. The branch was then placed onto a horizontally-oriented Lastolite 30 cm 18% gray exposure card with a 2 cm focusing target circle in the middle. The twig was rotated so that the larva was flat against the gray card. Larvae were then observed for 30 s to ensure that they remained still before a photograph was taken. From the images, we determined how many larvae in each experimental group were resting in a twig-like posture (rear claspers in contact with the branch and body raised away from it), and how many rested flat against the branch (rear claspers and front true legs in contact with the same branch). We then used the angle tool in Image J to measure the resting angle of the larvae in twig-like postures (see materials and methods for further detail on Image collection and analysis).

**Figure 4:**
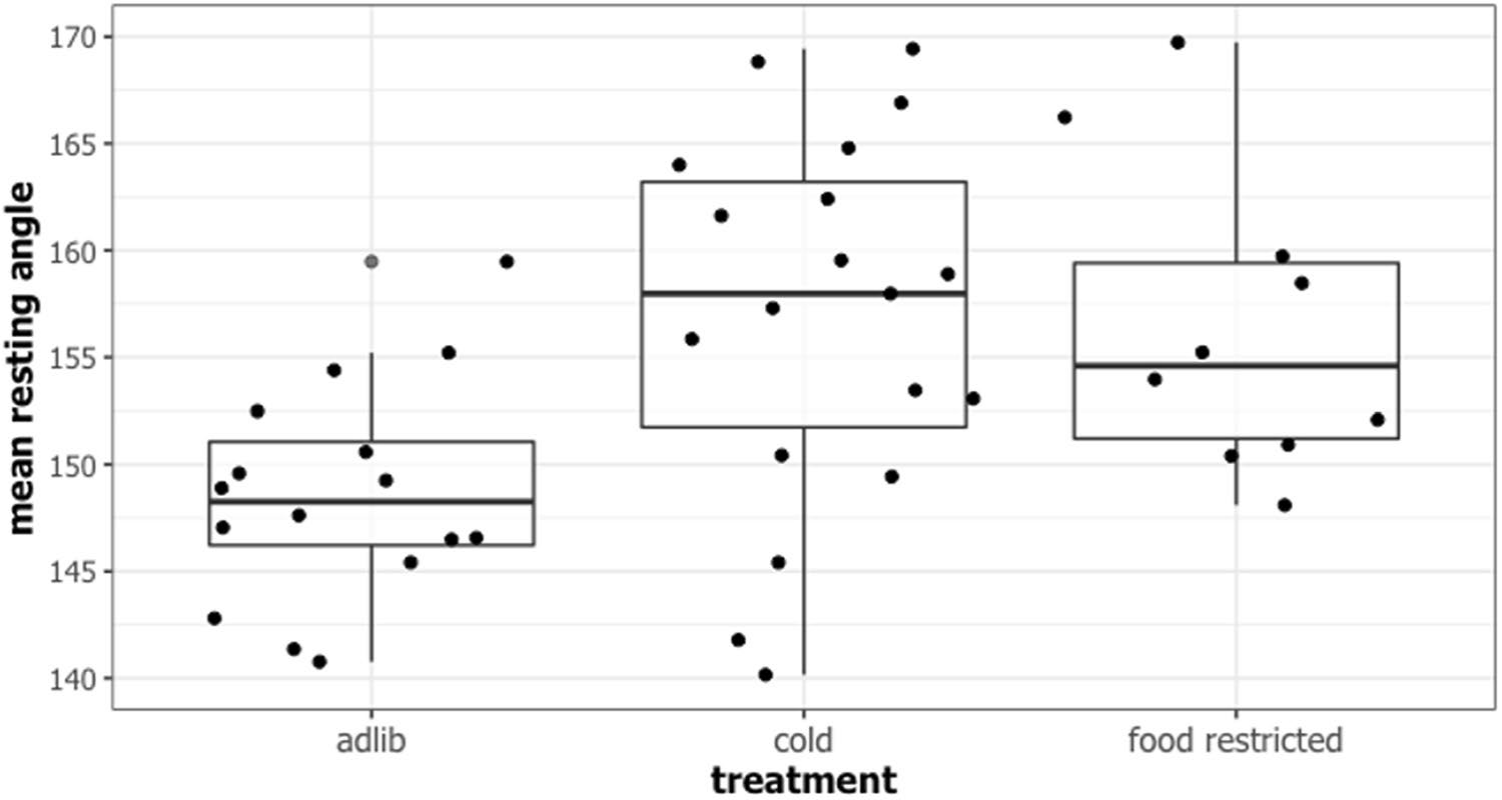
Resting angle of larvae in ad lib, cold, and food restricted conditions. The individual data points are plotted for each treatment, and each point represents the two resting angle measurements taken by two independent observers for a single larva.

We found that 82% of the ad lib larvae rested in a twig-like posture, compared with 71% of the chilled larvae and 42% of the food restricted larvae. Thus, while restricted larvae were significantly less likely to adopt a twig-like posture than ad lib larvae (Fisher’s test *p* = 0.0029), this was not true of chilled larvae (Fisher’s test *p* = 0.3829). Taken together with the findings of experiment 1, this suggests that food restriction may make caterpillars more vulnerable to predators. Furthermore, and in line with our predictions, when considering only those larvae that rested in a twig-like posture, both chilled (estimate 13.193 ± 3.195; t = 4.129, *p* = 0.000118; see Fig. 4) and food restricted larvae rested at a significantly more acute angle, with their heads closer to the branch (estimate 13.453 ± 3.837; t = 3.506, *p* = 0.000885).

## Discussion

We show that American peppered moth larvae gain antipredator benefits from postural camouflage: chicks took longer to attack caterpillars resting at an angle than those resting flat against a branch. This is the first species that has been conclusively demonstrated to benefit from postural camouflage, and our findings highlight the need to take the behavior of camouflaged prey into account when attempting to quantify their level of concealment. We also demonstrate that postures involved in concealing larvae are influenced by both food availability and ambient temperature, with food restriction causing significantly fewer larvae to rest at an angle, and both manipulations causing more acute resting angles in those that chose to rest at an angle. Since SI experiment 1 shows that caterpillars under standard conditions rest at significantly more acute angles than the twigs they resemble, this reduced resting angle in cold or food deprived larvae will further increase this difference compared to ad lib larvae. If predators perceive the difference, this could make caterpillars appear less twig-like, and reduce the benefit of postural camouflage. Such a hypothesis could be tested by comparing the time taken for predators to attack caterpillars resting at a range of angles.

There are several mechanisms via which caterpillars’ postures could confer anti-predator benefits. American peppered moth larvae are known to benefit from both masquerade and crypsis: predators mistake these larvae for twigs of their host plants, and find larvae difficult to detect when they are viewed against a background of similar-looking twigs^9,31,32^. Postural camouflage ensures that the resting angle of the larvae is more similar to that of the surrounding twigs, effectively enhancing the similarity in the context in which twigs and larvae are found. Since masqueraders are more likely to be mistaken for their models when found in the same context as their models^33^, it seems likely that twig-like postures of larvae serve to enhance the benefit of masquerade. However, this is unlikely to be the whole story since 10 of the chicks presented here with larvae resting flat (compared to 2 of the chicks presented with larvae in twig-like postures) directed their first pecks toward the larva. In this situation larvae cannot have been benefiting from masquerade as predators had no experience with inedible models^2,31^. It thus seems likely that resting at an angle also serves to exploit the perceptual processes of predators. In order to filter stimuli quickly and effectively, some species have evolved innate tendencies to use specific features (including posture) to initially classify items as potential prey^34^. Consequently, resting in a posture not commonly observed in prey could lead predators to classify larvae as non-prey items. Twig-like postures may also enhance crypsis. While it may seem counter-intuitive that a posture that exposes more of a larva’s body to view could enhance crypsis, there is reason to believe that this could be the case. In order for an animal to perceive an object, its nervous system must bind the features of that object into a coherent representation^35–37^. When larvae rest at an angle, predators may perceive the branch containing the larva as a single object given the similarity between the postures of larvae and the other features of the object (i.e. the twigs). In contrast, when larvae are resting flat, predators may perceive two distinct objects. This is how caterpillars in these positions appear to us, but this remains to be tested with predators.

Although postural camouflage provides obvious benefits, our findings hint at the possibility that this could come at an energetic cost. Depriving larvae of food resulted in fewer individuals resting at an angle, and both food restricted and chilled larvae rested closer to the branches. This is consistent with the idea that energy is required to hold a twig-like posture, a theory that could be tested more directly by measuring the energy consumption of larvae in different resting postures. As a result, energetically-stressed larvae may be unable to adopt steeper resting angles or may opt not to adopt these positions if the cost of doing so outweighs the benefit. These explanations could be teased apart by simulating predation on energetically-stressed larvae: if energetically-stressed larvae are unwilling (rather than unable) to rest in twig-like postures, simulated predation should cause them to rest at a steeper angle by enhancing the net-benefit of the posture (assuming that the antipredator benefit of postural camouflage is positively correlated with the degree of similarity between the angle of caterpillars and twigs).

It is unclear whether energetic costs associated with postural camouflage are likely to be widespread. Most, if not all, of the postures thought to serve this function involve holding the bodies in unusual positions. For example, cuttlefish (*Sepia officinalis*) hold their arms parallel to the body axis in a resting position, but will angle them obliquely or perpendicularly to match background objects^24^; orb-weaving spiders in the family Uloboridae (genus Tangaroa) usually rest with all of their legs spread out and equally flexed^38^, but individuals in the genus Uloborus are thought to use postural camouflage and instead hold their legs together in the same axis in a stick-like posture^38^. While these postures may at first appear associated with energetically-costly muscular activity, it is also possible that in some species they consume little if any extra energy. Certain smooth muscles, known as catch muscles, are able to reduce energy consumptions when holding fixed postures^28^. Catch muscles have not, to our knowledge, been found in lepidopteran larvae, and little is known about their use in maintaining defensive postures. Energetic costs are unlikely to be the only costs associated with postural camouflage. There may also be ‘loss of opportunity’ costs if the postures adopted prevent or restrict locomotion, or reduce foraging and/or hunting efficiency. Both masquerade and crypsis could also restrict animals to microhabitats that match their appearance^9,39^. Furthermore, acquiring, synthesizing, mobilizing and maintaining the pigments used in camouflaged patterns is likely to be costly^40^, although the latter is likely true of any color-based defensive strategy. Very little is yet known about any of these putative costs, limiting understanding of the evolution of camouflage.

In conclusion, we show that postural camouflage confers antipredator benefits, and that these postures are influenced by both food restriction and reduced ambient temperature. Our results suggest that if predators perceive these state-dependent postural changes, environmental conditions could potentially play an important role in determining the efficacy of postural camouflage, and provide some limited support for the idea that postural camouflage may be energetically costly in some species.

## Materials and methods

### Experiment 1

On day 2 of life, 32 chicks that had been trained to forage in the experimental arena (see SI Materials and Methods), but with no prior experience of either branches or live prey, were randomly divided into two groups of 16. Each chick then participated in three consecutive trials in which it was food restricted (but not water restricted) for 90 min before being placed in the experimental arena, where it encountered a single American peppered moth larva presented on a 20 cm long willow branch that contained 8 twigs. The twigs were cut to 5–6 cm in length to ensure that they were of similar size to the larvae. The testing cage had two separate sections: the buddy arena which housed companion chicks, measuring 20 cm × 50 cm × 50 cm; and the experimental arena, measuring, 100 cm × 50 cm × 50 cm. The branch was placed 15 cm from the buddy arena and the chick was placed 30 cm from the branch. A stop watch was started when the chick’s feet touched the floor of the experimental arena, and the time taken to attack the larva was recorded. In each trial, chicks were allowed unlimited time to make their first attack on the branch or larva, and the first object attacked was recorded. Chicks were then observed for a further 3 min to determine whether they ate the larva. Chicks were tested in a different random order in each trial.

The posture of the larva differed between our experimental groups: one group encountered larvae resting in a twig-like posture, and the other encountered larvae resting flat against the branch. To obtain larvae in each of these postures, we took advantage of natural variation in resting behavior. Forty-eight hours prior to the experiment, we took final instar larvae that had been group-reared in the laboratory in 2011 (see SI Materials and Methods), and transferred each larva into a separate 17 cm × 12 cm × 7 cm transparent plastic food box, with the lid punctured to provide ventilation. Larvae were transferred to reduce interference from other larvae that might affect resting position. Food (willow branches with leaves) was replenished daily to avoid the leaves drying out. On the morning of the predation experiment, each larva was transferred to an experimental branch inside an identical food box, and left for 1 h. Larvae were recorded as either resting at an angle or resting flat against the branch, they were then placed in a refrigerator for 10 min before use in the experiment in order to reduce their tendency to move. Prior to each trial, a box containing a larva in the required position was removed from the refrigerator and the branch it rested on was carefully placed in the experimental arena. Larvae were considered to be resting at an angle when their rear claspers were in contact with the branch and their bodies was raised away from the branch so that the front true legs were not in contact with the branch; they were considered to be resting flat against a branch when their rear claspers and front true legs were both in contact with the same branch. After waiting 2 min to ensure that the larva did not move, the experimental chick was introduced into the experimental arena. If the larva moved during these 2 min, the protocol called for a replacement larva to be used. None of the larvae moved during these 2 min, or prior to being attacked.

We analyzed the first peck data with a Generalized Linear Mixed Model with a binomial distribution and a logit link function using the package lme4^41^, and attack latency with a Generalized Linear Mixed Model with a negative binomial error structure using the package glmmTMB^42^.

### Experiment 2

Ninety-four final instar American peppered moth larvae from a single family were reared in the laboratory in 2012 (see SI Materials and Methods). We randomly divided the larvae into three experimental groups and manipulated their energetic state over a 48-h period. During this period, ad lib larvae (n = 28) were maintained at room temperature with a constant food supply, food restricted larvae (n = 31) were maintained at room temperature with no access to food, and chilled larvae (n = 35) were refrigerated ($\approx$ 1–4 °C) but had access to a constant food supply. These conditions were chosen as they approximate the most energetically-stressful conditions larvae are likely to experience in natural settings. For example, larvae could experience similar periods of food restriction if they fell from their host-plant or experienced poor weather that prevented foraging, and they could experience similar temperatures on the coldest days at the northern edge of their distribution. Furthermore, similar periods of food restriction have been shown to reduce larval growth and affect adult morphology in other species of lepidoptera^43,44^, and HMR’s personal experience of rearing this species of moth suggests that periods of food restriction or chilling > 48 h increases the mortality of peppered moth larvae (see also Ref.^45^). Moreover, after 48 h of food restriction the caterpillar gut has been shown to be clear of plant material (see also Ref.^46^), and post-hoc analyses of our data show that our larvae grew more slowly when chilled (see SI Fig. S3).

Larvae were housed in small groups (n = 4–6) in 279 mm × 159 mm × 102 mm clear plastic boxes that contained either branches with leaves (ad lib and chilled) or leafless branches (food restricted). After 48 h, larvae were transferred to a leafless branch and allowed to settle for 1 h. The branch was then moved by hand and placed onto a horizontally-oriented Lastolite 30 cm 18% gray exposure card with a 2 cm focusing target circle in the middle. Larvae were observed for 30 s to ensure that they remained still before a photograph was taken using a Canon 350D camera with a Canon zoom lens (EFD 18–55 mm). The camera was placed on a tripod with the camera pointing directly down. From the images, we determined how many larvae in each experimental group were resting in a twig-like posture, and how many rested flat against the branch (defined as above). We then used the angle tool in Image J to measure the resting angle of the larvae in twig-like postures. This allows users to measure an angle defined by three points. Using the circular scale of the gray card as a reference, the three points needed to measure the angle were delineated as follows: two centimeters along the branch, the midpoint between the two rear claspers of the larva, and the center of the larva’s head-capsule (see SI Fig. S1).

We analyzed the percentage of larvae that rested flat or in a posture with a Fisher’s exact test, and the angle of posture with a Generalized Linear Model with a Gaussian distribution and an identity link function. All analyses were conducted using R version 3.6.0^47^.

### Ethical note

This study was conducted following ASAB/ABS's Guidelines for the treatment of animals in behavioural research and teaching^48^ and following guidelines to the operation of the Animal (Scientific Procedures) Act 1986^49^. The nature of the study meant we did not require a U.K. Home Office License. This study was approved by the University of Glasgow’s Named Animal Care and Welfare Officer. At the end of the experiment all chicks were euthanized following Home Office schedule one methods (cervical dislocation).

## Supplementary information

Supplementary Information 1.

Supplementary Information 2.

Supplementary Information 3.

Supplementary Information 4.

Supplementary Information 5.
