# Supplementary material for: The antipredator benefits of postural camouflage in peppered moth caterpillars
Source: Sci Rep. 2020 Dec 10;10:21654. doi: 10.1038/s41598-020-78686-4 (PMC7728781; doi:10.1038/s41598-020-78686-4)
Supplement: Supplementary file 1 — Supplementary Information 1. [file 41598_2020_78686_MOESM1_ESM.docx]

Supplementary Information for

The antipredator benefits of postural camouflage in peppered moth caterpillars

Hannah M. Rowland, Robert P. Burriss, John Skelhorn

Hannah M. Rowland

Email: hrowland@ice.mpg.de

**This PDF file includes:**

Supplementary text 2100 words

Figures S1 to S3

Legend for Datasets S1-S4

SI References

**Other supplementary materials for this manuscript include the following:**

Datasets S1 to S4

**SI Materials and Methods**

**Chick Housing and Training**

***Housing:*** Fifty male chicks (Hyline strain) were obtained from a commercial hatchery in Preston, Lancashire, on the day of hatching. Chicks were housed at the University of Glasgow, in white metal cages measuring 120cm x 50cm x 50cm. One cage housed the experimental chicks (n=32), and the second housed buddy chicks (n=18). Each cage was heated to 27^o^C, following guidelines to the operation of the Animal Scientific Procedures Act 1986 (1), using either one Interbrooda standard (40cm x 60cm) or two Interbrooda mini (40cm x 40cm). These brooders, also known as ‘electric hens’, consist of an electrically heated square or rectangular plate which stands on four adjustable legs. This enables the height and temperature of the plate to be adjusted as the chicks grow. The laboratory was held at a constant temperature of 24 degrees. Temperatures beneath the brooders and the ambient room temperature were monitored and recorded daily. Water was provided *ad libitum* in two one liter ‘jam-jar’ drinkers in each cage. Brown chick starter crumbs were also provided *ad libitum* in each cage in two ceramic food bowls that contained a clear plastic cylinder to prevent chicks sitting in the food. The cages were lined with brown paper cage liners, which were replaced daily. During training and experimenting, short periods of food restriction were necessary to ensure chicks were motivated to forage. During all periods of restriction, chicks had access to water but not food. All restriction periods were in accordance with UK Home Office regulations and guidelines and were no longer than 90 minutes.

Chicks were subject to a 14:10h light:dark cycle and the lighting had no UV component. All subjects were marked with identifying color codes on the top of their heads with non-toxic Sharpie marker pens. Markings did not result in any aggressive behavior between individuals. Weights were monitored for welfare purposes throughout the experiment, with all experimental chicks gaining as much weight as buddy chicks (who experienced fewer periods of food deprivation) as the experiment progressed. The experiment was conducted following guidelines to the operation of the Animal (Scientific Procedures) Act 1986 (1). The nature of the study meant we did not require a U.K. Home Office License. At the end of the experiment all chicks were euthanized following Home Office schedule one methods (cervical dislocation).

***Training:*** On arrival at the laboratory chicks were allowed to acclimatize for three hours, after which food was removed from all of the cages for one hour. After one hour of food deprivation, chicks were trained to forage alone in the experimental arena. Without such training, chicks placed in the arena alone become distressed, call loudly, and do not eat. Training was conducted in three identical experimental cages simultaneously. These cages were similar to the chicks’ home cages, except that a mesh divider was used to create two separate sections: the buddy arena, measuring 20cm x 50cm x 50cm; and the experimental arena, measuring, 100cm x 50cm x 50cm. There was no brooder in these cages, and the floor was covered with the backing paper of sticky-backed plastic (a waxy paper imprinted with a faint red grid whose intersections were at 2.5cm intervals). All chicks were given six, four-minute training sessions, during which they were presented with brown chick starter crumbs scattered on the floor of the experimental arena. In trials one and two, chicks were placed in the experimental arena in groups of three; in trials three and four, chicks were placed in the arena in pairs. In trials five and six, lone chicks were placed in the arena. In all training trials (and all subsequent test trials), three buddies were placed in the buddy arena. Buddy chicks had free access to food and water in their home cage, but only access to water during training and experimental trials. Buddy chicks serve as visual companions to the experimental chicks during the foraging experiment, thereby reducing any potential distress from placing experimental chicks in the arena alone. Buddy chicks were used on a rotational basis, and were changed every three trials or after one hour, whichever came first. They never had access to the experimental part of the arena, and never interacted with branches or larvae. By the end of pre-training, all experimental chicks were eating brown starter crumbs from the floor of the arena without any signs of distress.

**Larva Housing**

*Biston betularia* larvae were reared in 2011 and 2012. In 2011, larvae were reared from eggs obtained from a clutch (c. 1000 eggs) that was laid by a single wild-caught female attracted to a light-trap in Cheshire, UK (care of Jon Delf). In 2012, larvae were reared from eggs obtained from crossing experiments between female offspring of the 2011 stock and males from laboratory stock at the University of Liverpool (care of Ilik Saccheri). Larvae were hatched and reared in the laboratory, at ambient temperature and were subject to natural lighting and day length. Larvae were fed on either white willow (*Salix alba;* in 2011) or gray willow (*Salix atrocinerea*; in 2012) collected from trees growing alongside the River Kelvin in Glasgow, UK (*S. alba*), or from various sites on the Wirral peninsula, UK (*S. atrocinerea*). First instar larvae were housed together in a 73 x 73 x 73 mm transparent plastic box with holes in the lid to provide ventilation. At this stage in their development, larvae produce large quantities of silk when movement around the food plant, and often become tangled which reduces their ability to feed. To prevent this, we used a round artist’s paintbrush to place larvae onto the foodplant and break up the silk twice daily. Food was replenished daily to avoid the leaves drying out. Second instar larvae were transferred by paintbrush into 17cm x 12cm x 7cm transparent plastic food boxes (each containing 100 individuals), with lids punctured to provide ventilation. When larvae reached instar three, they were either housed in groups of 20 in containers of the same size (2011), or kept in groups of approximately 100 but were moved into 16L plastic boxes (2012). Larvae were maintained in these boxes until they reached their final instar. Boxes were cleaned daily between 18:00 and 22:00 hours in 2011 and 10:00 and 14.00 hours in 2012 using laboratory tissue paper to remove condensation and frass. Once larvae were third instar they were big enough to move by hand, and were moved off the old food by gently dislodging the rear claspers from the branch. Food was replenished daily.

**SI Experiment 1**

**Do larvae and twigs rest at similar angles?**

Research indicates that American peppered moth larvae rest at angles similar to, although not quite as high as, twigs of their host plant (2). However, measurement techniques have improved in the time since this study was published. We therefore compared the angle at which larvae rested on white willow and gray willow, and the angles of the branches of each tree species.

***Material and Methods:*** Final (5^th^) instar larvae were photographed using a Canon 350D digital camera. Photographs were taken on three dates in July 2011 and two dates in August 2012 during normal husbandry practices. Each larva was photographed once, and then moved to a housing container. Branches to which larvae were clasping with their body held still, rigid, and away from the branch (i.e. in a twig-like posture; see main paper Figure 1) were moved by hand (HMR or RPB) onto a piece of horizontally-oriented gray standard photographic card with a 2cm diameter circle printed in the middle. Larvae were observed by both HMR and RPB for 30 seconds to ensure that they remained still and rigid before a photograph was taken. Any larvae that moved during transferal, or when on the gray card, were returned to the housing container without being photographed. Using these images, the resting angles of 142 larvae were measured by two independent persons: RPB and HMR measured the resting angle of 57 larvae in 2011, and RPB and HMR measured the resting angle of 83 larvae in 2012. The resting angles were measured using the angle tool in Image J. Using the circular scale of the gray card, the three points needed to measure angle were delineated as follows: two centimeters along the branch, the midpoint between the two rear claspers of the larva, and the centre of the larva’s head-capsule (see Figure s1). We also collected 63 branches of white willow and 24 branches of gray willow for comparison to larvae. We removed the leaves and cut the twigs 5-6cm from the main stem. Twigs were photographed with the same camera against the same standard photographic card. Twig angles were measured with the angle tool in Image J by delineating three points: 2cm along the branch, the center of the base of the twig, and 2cm up the midline of the twig.

***Statistical analyses:*** Measurement reliability was assessed using intra-class correlation using the package IRR (3). We compared the resting angles of larvae reared on white willow to larvae reared on gray willow, and twigs of white willow to twigs of gray willow using a generalized linear model with a Gaussian distribution and an identity link function.

***Results:*** The intraclass correlation coefficients for the angle measurements taken by the two observers were as follows: 0.97 (F_(56,57)_ = 66.3 , p = 4.81e-37) for larvae reared on white willow, 0.94 (F_(82,73.5)_ = 33.4 , p = 2.66e-37) for larvae reared on gray willow, 0.987 (F_(62,62.1)_ = 147 , p = 7.52e-51) for white willow twigs and 0.874 (F_(23,7.29)_ = 21.9 , p = 0.000127) for gray willow twigs. We calculated a mean of the two measurements for each larva/twig and used this in all subsequent analyses. The angle of white willow and gray willow twigs did not differ (estimate = 6.748 +/- 3.533, t = 1.91, p = 0.0595), but larvae rested at significantly more acute angles than the twigs, i.e., with their heads closer to the branch, than the twigs grew (estimate = -0.178306 +/- 0.020036, t = - 8.899, p = < 2e-16 ; see Figure s2). Intriguingly, larvae reared on gray willow rested at more obtuse angles than larvae reared on white willow (estimate = -8.0444 +/- 1.4994, t = -5.365, p = 3.33e-07), meaning that the difference in resting angle between twigs and larvae was larger for larvae reared on gray willow (tree type x larva interaction: estimate = 0.106038 +/- 0.025017, t = 4.239, p = 3.29e-05). Taken together our findings clearly show that, while larvae that assume twig-like postures match the ‘posture’ of twigs more closely than larvae that rest flat against a branch, larvae do not closely match the angles achieved by twigs. This failure to closely mimic the orientation of twigs to the branches may be explained by the presence of a physiological constraint that means that larvae are unable to adopt the higher angles that would be advantageous, there being a cost to adopting this posture which increases with resting angle such that the cost of resting at a higher more twig-like angle outweighs the antipredator benefits; or that resting angle (at least beyond a certain value) does not improve the anti-predatory benefits of twig mimicry.

**SI Experiment 2**

**How common it is for larvae to rest at an angle?**

***Materials and Methods:*** In September 2012, 433 larvae from ten different families were used to determine the proportion of individuals resting in a twig-like posture. During routine husbandry, we located each larva that was clasping a willow branch and noted whether it was resting at an angle in a twig-like posture or resting flat again the branch. Resting at an angle was defined as resting with the rear claspers in contact with the branch and the body raised away from the branch so that the front true legs were not in contact with the branch, and resting flat against a branch was defined as the rear claspers and front true legs both being in contact with the same branch.

***Results:*** Under ad libitum conditions, and across the ten different families measured, 68% of the larvae rested at an angle, whereas 32% rested flat against the branch. Thus, significantly more of the larvae rested at an angle than rested flat (binomial test p = 0.0054). This indicates that the behavior is common, but not ubiquitous.

**
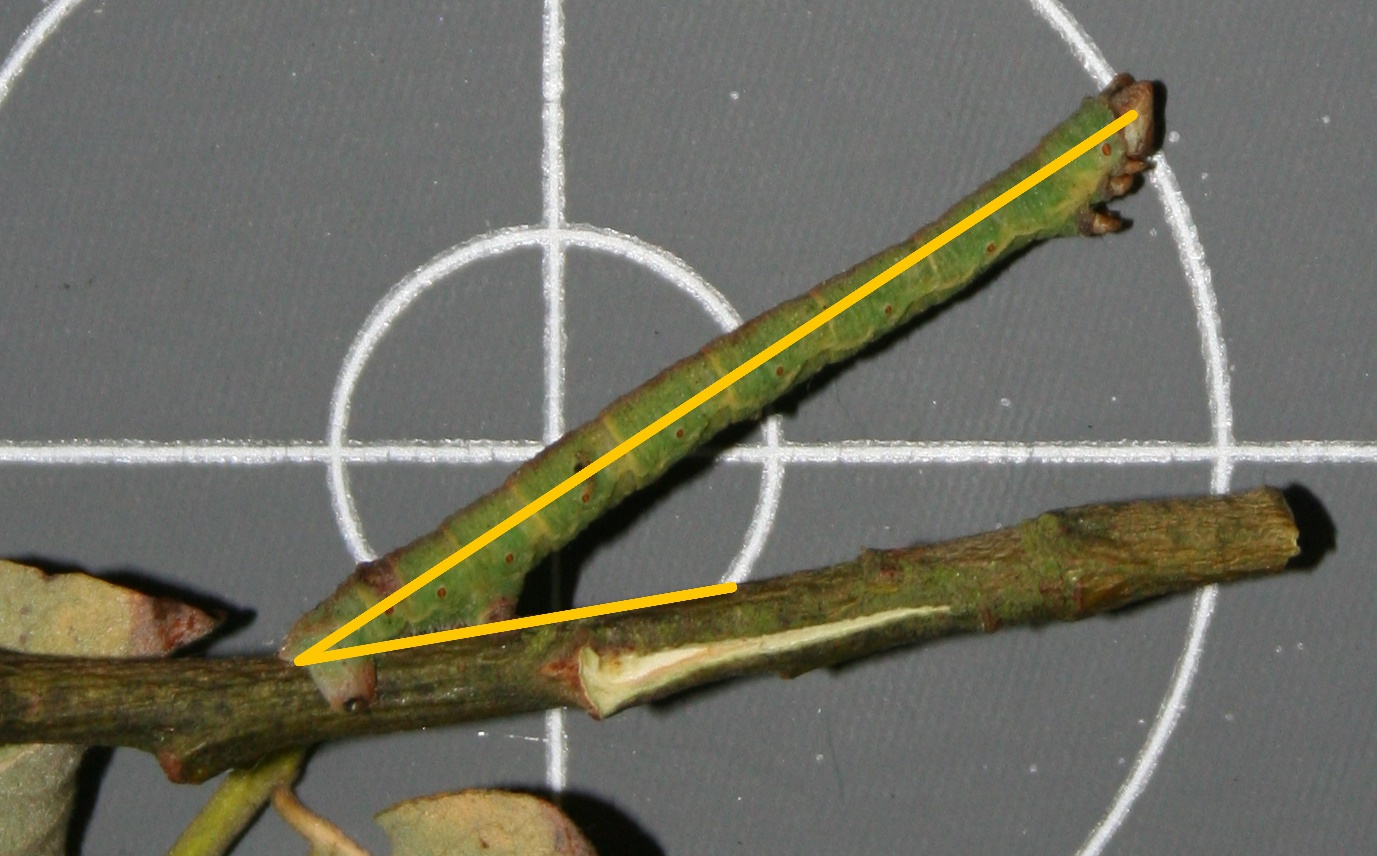
**

Fig. S1. points for angle measurements

**
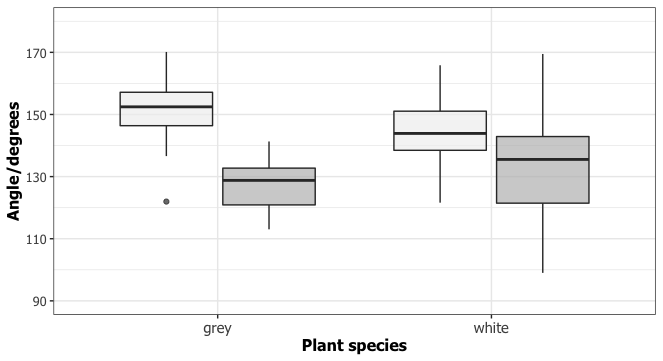
**

Fig. S2. the angle of larvae (light gray) and twigs (dark grey) found on gray willow and white willow branches.


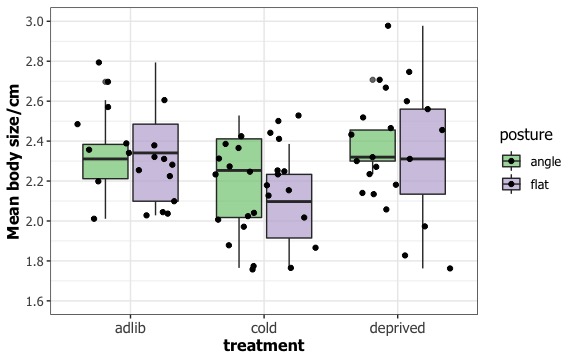


Fig. S3. The body size of caterpillars in the three treatments that rested at an angle, or rested flat against the branch.

Dataset 1 (separate file) Experiment one data. Chick ID colour code (three letters), Chick ID (numeric), treatment (p = posture, f = flat), Trial (number 1-3), identity of first peck (b = branch, c = caterpillar), Time to peck larva in seconds, larvae eaten (y = yes, n = no)

Dataset 2 (separate file) Experiment two data. Treatment (deprived, adlib, cold), angle of larva

Dataset S1 (separate file) Experiment S1. the angle of larvae and twigs for gray willow (grey) and white willow (white) branches.

Dataset S2 (separate file) Experiment S2. Family identity of larvae, the number resting flat and at an angle in each family.

**SI References**

1. U. K. Government, Guidance to the operation of the Animals (Scientific Procedures) 1986. ScotPIL manual - avian species. (2009).

2. M. Dockery, J. Meneely, P. Costen, Avoiding detection by predators: the tactics used by Biston betularia larvae. *British Journal of Entomology and Natural History* **22**, 247-253 (2009).

3. M. Gamer, J. Lemon, I. Fellows, P. Singh (2019) irr: Various Coefficients of Interrater Reliability and Agreement. (https://www.r-project.org).
